# PRRT: identikit of the perfect patient

**DOI:** 10.1007/s11154-020-09581-6

**Published:** 2020-09-25

**Authors:** M. Albertelli, A. Dotto, C. Di Dato, P. Malandrino, R. Modica, A. Versari, A. Colao, D. Ferone, A. Faggiano, on behalf of NIKE

**Affiliations:** 1 Endocrinology Unit, IRCCS Ospedale Policlinico San Martino, Genova, Italy; 2 Endocrinology Unit, Department of Internal Medicine and Medical Specialties (DiMI) and Center of Excellence for Biomedical Research, University of Genova, Genova, Italy; 3 Endocrinology, Department of Experimental Medicine, “Sapienza”, University of Rome, Rome, Italy; 4 Endocrinology, Department of Clinical and Experimental Medicine, Garibaldi-Nesima Medical Center, University of Catania, Catania, Italy; 5 Endocrinology, Department of Clinical Medicine and Surgery, “Federico II” University of Napoli, Napoli, Italy; 6 Nuclear Medicine, Azienda Ospedaliera Santa Maria Nuova-IRCCS Reggio Emilia, Reggio Emilia, Italy; 7 Depart. of Experimental Medicine, Division of Medical Physiopathology Sapienza University of Rome Viale del Policlinico 155, 00161 Rome, Italy

**Keywords:** Neuroendocrine tumors, Therapeutic sequence, Peptide receptor radionuclide therapy, Predictor, Response

## Abstract

Peptide receptor radionuclide therapy (PRRT) has been strengthened since the publication of NETTER-1. Nevertheless, the correct positioning in the therapeutic algorithm is debated, and no optimal sequence has yet been standardized. Possible criteria to predict the response to PRRT in neuroendocrine tumors (NET) have been proposed. The aim of this review is to define the perfect identity of the eligible patient who can mostly benefit from this therapy. Possible predictive criteria which have been analysed were: primary tumor site, grading, tumor burden, FDG PET and ^68^Ga-PET uptake. Primary tumor site and ^68^Ga-PET uptake do not play a pivotal role in predicting the response, while tumor burden, FDG PET uptake and grading seem to represent predictive/prognostic factors for response to PRRT. The heterogeneity in trial designs, patient populations, type of radionuclides, previous therapies and measurement of outcomes, inevitably limits the strength of our conclusions, therefore care must be taken in applying these results to clinical practice. In conclusion, the perfect patient, selected by ^68^Ga-PET uptake, will likely have a relatively limited liver tumor burden, a ki67 index <20% and will respond to PRRT irrespective to primary tumor. Nevertheless, we have mostly prognostic than predictive factors to predict the efficacy of PRRT in individual patients, while a promising tool could be the NETest. However, to date, the identikit of the perfect patient for PRRT is a puzzle without some pieces and still we cannot disregard a multidisciplinary discussion of the individual case to select the patients who will mostly benefit from PRRT.

## Introduction

Over the past 2 decades, peptide receptor radionuclide therapy (PRRT), has been proved to be an effective and safe therapeutic option in patients with inoperable or metastatic well-differentiated neuroendocrine tumors (NETs) [8, 32, 34, 40, 41, 46]*.*

Somatostatin receptor ligand (SrL) labelled with Indium-111 (^111^In-DTPA-Octreotide) was the first radiopharmaceutical applied with encouraging results in terms of symptomatology. Nevertheless, objective responses were rare, while hematological side effects were also observed [40]. Subsequently, new analogues labelled with the β-emitting radionuclides Lutetium-177 and Yttrium-90 were introduced. In the following 15 years, uncontrolled trials on both radiopharmaceuticals in different types of NETs reported disease-control rates (DCR)s of 68–94% and a significant increase of overall survival (OS) and progression free survival (PFS) ([8, 13, 34], Kwekkeboom et al. 2005)*.* Furthermore, biochemical and symptomatic responses, and quality of life improvement have been reported [36].

Data about safety of PRRT are comforting. The most frequent acute side effects are nausea and vomiting, fatigue, abdominal pain and myelosuppression, that are generally mild, self-limiting and reversible. Carcinoid crisis is very rare. Kidney damage is a long-term side effect, but renal failure can be prevented by the coadministration of positively charged amino acids. Haematological toxicity, such as leukemia or myelodysplastic syndromes has been reported in less than 5% of patients who received PRRT [47].

Despite this long-time experience, for many years data about efficacy and safety of PRRT derived only from few early-phase trials or retrospective studies, until the publication of the recent first randomized phase III NETTER-1 trial [74]**,** comparing PRRT (^177^Lu-DOTATATE) to high-dose octreotide LAR in patients with progressive inoperable or metastatic midgut NETs. The objective response rate (ORR) was 18% in the ^177^Lu-DOTATATE group versus 3% in the control group (*p* < 0.001). The median progression-free survival (mPFS) was not reached in the ^177^Lu-DOTATATE group and it was 8.4 months in the control group (hazard ratio for disease progression or death with ^177^Lu-DOTATATE vs. control, 0.21; 95% CI, 0.13 to 0.33; *p* < 0.001), which represented a 79% lower risk of disease progression or death in the ^177^Lu-DOTATATE group than in the control group. In addition, the interim analysis indicated that the estimated risk of death was 60% lower in the ^177^Lu-DOTATE group than in the control group (hazard ratio 0.40; *P* = 0.004). These impressive results, seen in the NETTER-1 study, substantiates the use of PRRT in NET patients.

However, no sequence is as yet standardized by major international guidelines and the correct positioning of PRRT in the therapeutic algorithm is up for discussion.

The antitumor effect of PRRT is based on the radiolabeled SrL ability to bind somatostatin receptors (SSTR), highly expressed in NETs [39]. A strong expression of SSTR-2 (Krenning Scale 3–4 as fulfilled in the NETTER-1 trial) seems to have an impact on the outcome, but also site of the primary tumor, tumor load, grading and the Positron Emission Tomography (PET) with ^68^Ga-DOTA-peptide and/or with ^18^F-fluorodeoxyglucose (18F-FDG) uptake may influence the efficacy of PRRT. Some prospective and retrospective studies analyzed these parameters individually, while the potential role in predicting response to PRRT of these factors has never been explored globally. Moreover, according to a recent experience of [9], high predictive and prognostic power on the outcome with PRRT, are observed for the “NETest”, a specific liquid biopsy which measures neuroendocrine tumor gene expression in blood and aims at defining the biological activity of an individual NET in real time. The achievement reported in the cited paper are promising, also regarding the prediction of response to PRRT in different types of NET.

Nevertheless, since the NETest is not widely available in clinical practice, and it has still to be tested and further validated in other studies, the identification of reliable predictors of tumor response to PRRT is still urgently needed, to improve the outcome of PRRT, providing directions in clinical decision-making, toward a more personalized therapy.

The aim of this review is to revise all potential predicting factors of response to PRRT, finally defining the perfect identity of the eligible patient who can benefit most from this therapy.

## Methods

We performed a literature search of MEDLINE (PubMed database and PMC) and Ovid to identify potentially relevant articles on the predictive factors of efficacy of PRRT. The search was last updated September 23rd 2019. The search strategy included the following terms: “neuroendocrine tumor”, “neuroendocrine carcinoma” and “peptide receptor radionuclide therapy”. Only articles published in the English language were considered. Additional studies were identified by reviewing the references of all selected articles. The methods of potentially eligible studies were assessed independently by five reviewers (MA, AD, CDD, PM, RM). Studies considered potentially eligible were retrieved in full-text and evaluated. Disagreements were resolved by consensus or referred to another reviewer for arbitration (AF).

### Eligibility criteria

Eligible studies investigated the use of either Lutetium-177 or Yttrium-90 or both in patients with histologically confirmed NET. Both retrospective and prospective studies were included; single-arm studies were also eligible. To be included, a study needed to report on participants treated with PRRT. Original articles were considered without any restriction, and Editorials and Letters were excluded. Reviews of PRRT were scrutinized for references to eligible trials, but were themselves excluded. Studies of Merkel cell carcinoma, phaeochromocytoma and medullary thyroid carcinoma were also excluded, as were reports with less than five patients or where there was no report on any of the considered endpoints. Studies that examined PRRT alone were selected, while studies in combination or sequence with other therapies (eg. use of radiosensitizing chemotherapy) were excluded. Case-reports and non-English texts were excluded as well. The selected abstracts were then further assessed for a full-text evaluation.

**Fig. 1:**
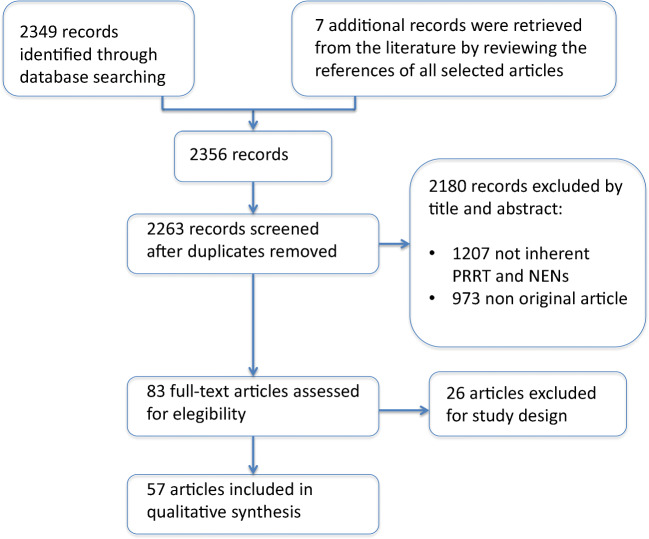
Study selection sequence to perform the review

Database searches yielded the following 2349 references: 626 from PubMed, 1003 from PMC, and 722 from the Ovid database. Seven additional references were retrieved from the literature. Exclusion of duplicates left 2263 records, and 2180 of these did not satisfy inclusion criteria. Full texts were examined in 83 publications concerning the use of PRRT in NETs, and 26 were excluded due to the study design. Thus, 57 papers were considered for qualitative analyses of PFS, OS and ORR (Fig. 1).

## Results

After selection of original articles from literature we recognized four parameters that more commonly were investigated as potential predictors of response to PRRT: primary, PET-uptake, tumor burden and grade (ki67 index). Some authors evaluated even performance status, that seems to have some degree of influence, however there are few data about this point, hence we decided to focus our attention on the four above stated criteria.

### Primary

In 26 papers (2007–2019), 8 prospective, 17 retrospective and one prospective/retrospective – German registry, primary tumor site was evaluated according to PRRT. Table 1 shows all selected original articles. The population comprised over 4050 patients with metastatic and almost all progressive NET, mainly gastroenteropancreatic. 177Lu was employed in 11 studies, 90Y in 2 and both in 13. Number of cycles ranged from 1 to 9 and cumulative dose varied usually between 14.7 and 30 GBq.

**Table 1:**
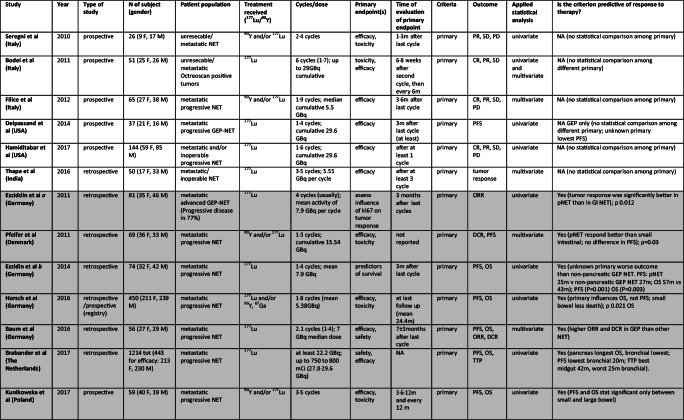

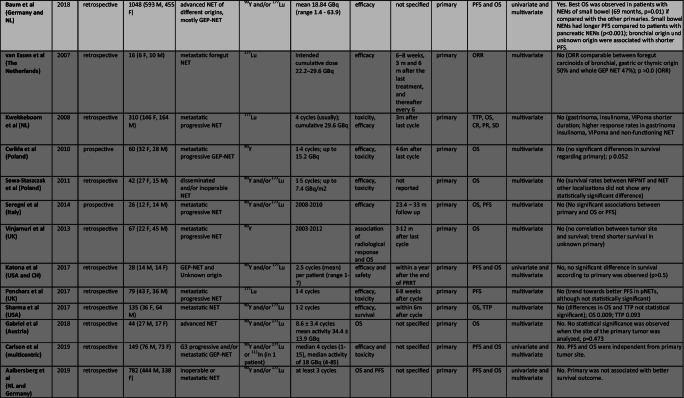
Studies assessing role of *Primary* as predictor of different outcomes after PRRT. In light gray “Positive studies”, in gray “Negative Studies”, white for only descriptive studies

Abbreviations: M, male; F, female; GEP, gastroenteropancreatic; SI, small intestinal; GI, gastrointestinal; NFPNT, pancreatic non functioning NET; pNET, pancreatic NET; NET, neuroendocrine tumor; GBq, Gigabecquerel; mCi, millicurie; ORR, objective response rate; PFS, progression free survival; OS, overall surviv TTP, time to tumor progression; DCR, disease control rate; DFS, disease free survival; DSS, disease specific survival; CR, complete response; PR, partial response; SD, stable disease; PD, progressive disease

In 20 studies the role of primary in PRRT was analyzed in detail [1, 4, 5, 12, 15, 21, 24, 27, 32, 35, 42, 46, 59, 60, 68, 70, 72, 78, 79], but primary showed an impact on response to PRRT only in 8 [4, 5, 12, 21, 24, 32, 42, 60]. No association was noted between positive and negative studies and patient populations, sample size, study design or radionuclide employed (177Lu or 90Y).

#### “Positive studies”

GEP NET showed higher PFS, ORR and DCR than other NET in the study by [4]. PFS in GEP was 30.3 months vs 17.4 months in the total NET population, ORR was 54.2% vs 40.6% and DCR was 100% vs 93.8%. Median OS was 34.7 months in both groups. Importantly, among patients with GEP NET undergoing >1 PRRT cycles, complete responders were 25%, the highest value reported and neither CR, nor PR in other NET were reported.

[12] reported longest median OS in panNET (71 months), and lowest in bronchial (52 months) and NET of unknown origin (53 months). Median PFS was 30 m in panNET, like NET of unknown origin (29 months) and 20 m in bronchial. Best time to progression (TTP) was reported in midgut NET (42 months), and worst in bronchial NET (25 months), while panNET had 31 m and NET of unknown origin 37 months .

Again, [5] in their retrospective analysis found that the site of origin of NENs was a predictor of median overall survival. Best OS was, in fact, observed in patients with NENs of small bowel (69 months, *p* = 0.01), which was statistically significant if compared with the other groups (pancreas, lung, other primaries, unknown origin). Even PFS was correlated with the site of primary tumor, having patients with small bowel NENs a longer PFS compared to patients with pancreatic NENs (*p* < 0.001); bronchial origin and unknown origin were associated with a significantly shorter PFS.

The site of primary influences OS, but not PFS in the study by [32], as patients with small bowel NET were significantly less likely to die (p 0.021).

Statistically significant difference in PFS and OS between large and small bowel NET was found by [42] (OS 82.5 vs 58.1 months; PFS 40.3 vs 29.5 months). [60] reported no significant difference in PFS among panNET (27 months), NET of unknown origin (30 months) and small intestinal NET (not reached at the time of analysis).

[21] show that ORR was significantly better (p 0.012) in panNET than in GI NET and in 2014 [24] reported worse outcome for NET of unknown origin than nonpancreatic GEP NET, having shorter PFS (*P* = 0.001) and OS (*P* = 0.003). PFS in panNET was 25 months vs nonpancreatic GEP NET (27 months) and OS was 57 vs 43 months.

In 2 studies, NET of unknown origin showed a trend of shorter survival [79] and the lowest PFS [18] without statistical significance.

#### “Negative studies”

Six studies [15, 17, 27, 35, 68, 79] reported no significant difference in survival according to primary. Comparable ORR between foregut carcinoids of bronchial, gastric or thymic origin (50%) and whole GEP NET (47%) was reported by [78], but time to progression (TTP) was shorter in foregut than in GEP NET.

[70] reported that TTP rates were not significantly different among pulmonary, small bowel NET and panNET (*P* = 0.093). The single result that achieved statistically significance was a longer OS in small bowel NET compared with other primary. In fact, OS after the first PRRT was 95.4, 37.3, and 20 months for small bowel, pancreatic, and NET of unknown origin, respectively (*P* = 0.009) [70].

A study by [1] showed conflicting results, founding difference in OS in the univariate analysis (small bowel NET had longer OS than pancreatic, lung and large intestine primaries) but not in the multivariate analysis. Reported median OS for non-functioning panNET was 25.7 months and for other NET was 46.7 months in the study of [72], but data were not statistically significant. A not significant trend towards a better PFS in panNETs was also reported by [59].

Regarding functionality, gastrinoma, insulinoma, VIPoma showed shorter response duration, and higher response rates were reported in gastrinoma, insulinoma, VIPoma, non-functioning NET than in carcinoid (p < 0.01), according to a sub-analysis conducted on a subgroup of patients from the study of Kwekkeboom et al. [46].

In 5 studies, no correlation was reported between primary and any outcome, [8, 25, 30, 67, 75].

### Pet

Ga-68 labeled somatostatin analogs and 18F-FDG PET have been evaluated as potential predictors of response to PRRT in twenty-one studies conducted from 2009 to 2019. Table 2 and 3 show all selected original articles where Gallium/FDG PET were evaluated as a predictive factor of response to PRRT. Overall, more than one thousand patients with NET were evaluated. Eight studies are prospective non-randomized clinical trials while thirteen studies are retrospective.

**Table 2:**
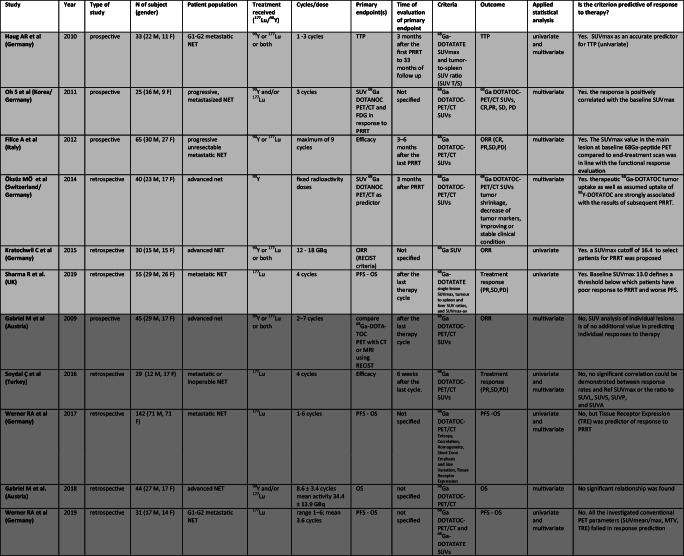

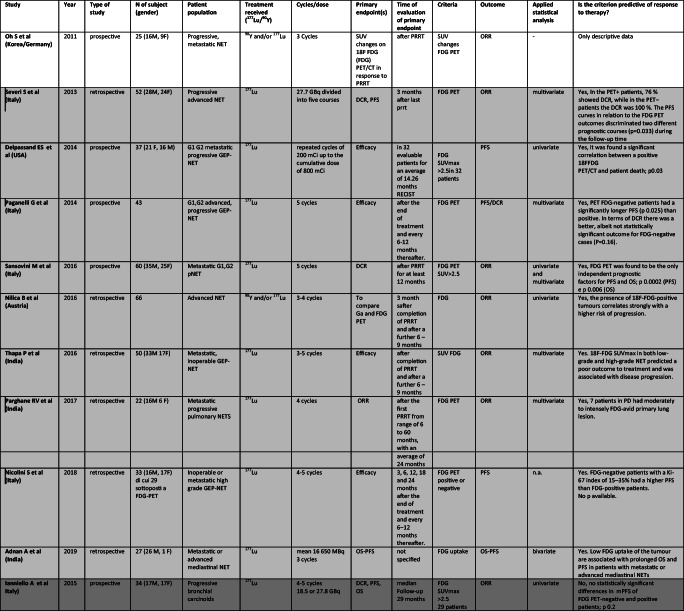
Studies assessing role of ^*68*^*Ga-DOTA-peptides PET*as predictor of different outcomes after PRRT. In light gray “Positive studies”, in gray “Negative Studies”

**Table 3:**
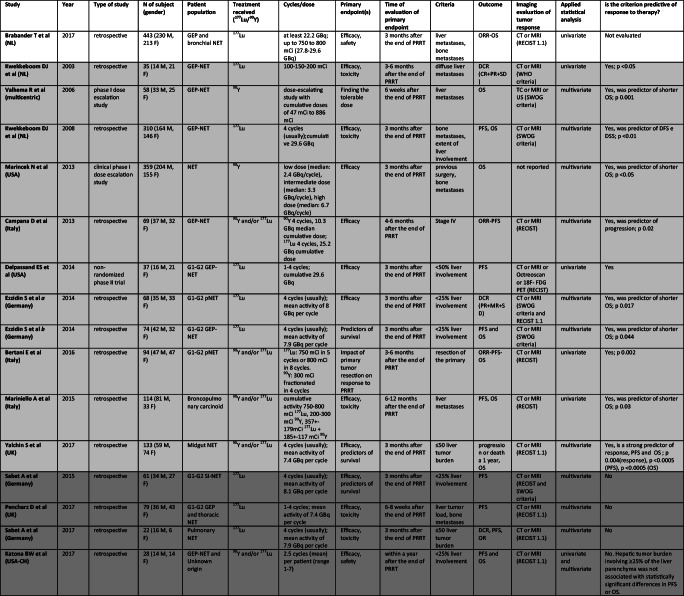
Studies assessing role of *Tumor Burden* as predictor of different outcomes after PRRT. In light gray “Positive studies”, in gray “Negative Studies”, white for only descriptive studies

Abbreviations: M, male; F, female; GEP, gastroenteropancreatic; SI, small intestinal; NET, neuroendocrine tumor; GBq, Gigabecquerel; mCi, millicurie; ORR, objective response rate; PFS, progression free survival; OS, overall survival; TTP, time to tumor progression; DCR, disease control rate; DFS, disease free survival; DSS, disease specific survival; CT, computed tomography; MRI, magnetic resonance imaging; US, ultrasonography; RECIST, Response Evaluation Criteria in Solid Tumors; SWOG, Southwest Oncology Group

Concerning ^**68**^**Ga-DOTA-peptide PET,** all studies assessed tumor standardized uptake value (SUV) as a quantitative parameter, to predict response to PRRT (see Table 2). Among eleven papers that analyze ^68^Ga- PET, three papers did not find any significant result that attributes a predictive role to maximum SUV (SUV_max_) during ^68^Ga-PET ([26, 27, 73]), two papers from the same group [80, 81] identified other parameters besides SUV_max_, able to predict outcome of PRRT treatment, and six identified SUV_max_ as a predictor of response to therapy [25, 31, 38, 53, 54, 71]. All studies were performed on rather small NET series. Both negative and positive studies comprise retrospective and prospective experience and no particular features characterize a group relative to the other. No association was noted between positive and negative studies and patient populations or radionuclide employed (177Lu or 90Y).

#### “Positive studies”

In detail, pre therapeutic SUV_max_ was identified as a predictor of TTP in a population of 33 patients [31]. However, the SUV_max_ was identified as a predictor of TTP in univariate analysis only (*p* = 0.04), while, according to the Cox proportional hazards model, authors identified another parameter (the change in SUV of the tumors relative to the maximal splenic uptake – Delta SUV T/S) as the only predictor of TTP in both univariate (*p* = 0.006) and multivariate analyses (*p* = 0.03) [31]. Öksüz et al. [54] showed that 68Ga-DOTATOC tumor uptake was strongly associated with the response to PRRT (*p* < 0.001). Furthermore, the authors identified a SUV cut-off of 17.9 to predict treatment response to PRRT. Similarly, [38], observed significant differences in the mean SUV_max_ for non-responding vs responding liver metastases in 30 NET patients, proposing a SUV_max_ cut-off of 16.4 to select patients for PRRT. Sharma et al. also investigated SUV_max_ and SUV_mean_ of ^68^Ga-PET/CT as possible predictor of PRRT response. Single lesion SUV_max_ was, in fact, predictive of both response to PRRT (*p* = 0.031) and PFS, and SUV_mean_ correlated with PRRT response (*P* = 0.039) as well. The authors also indicated a cut-off of 13.0, when considering single lesion SUV_max_, to give high sensitivity and specificity in prognosticating a favorable treatment response. Tumor to liver SUV ratio (SUV_T/H_) and tumor to spleen SUV ratio SUV_T/S_ were investigated as well, but neither SUV_T/S_ or SUV_T/H_ were predictive of response to PRRT [71]. A significant correlation between the ORR to PRRT and the ^68^Ga-DOTANOC PET baseline SUV_max_ was also found in the prospective trial conducted from **[**53**]**, but p value was not reported. Similarly, [25] showed that the SUV_max_ in the main lesion at baseline was predictive of response as indicated by functional evaluation.

#### “Negative studies”

On the contrary, in a multicenter study [80], both SUV_max_ and SUV_mean_ of ^68^Ga-PET/CT failed in response prediction to PRRT. However, the authors identified other textural features representing intratumoral heterogeneity**,** such Tissue Receptor Expression (TRE), able to predict OS (p = 0.003) and PFS (p = 0.02). Similar results were found by the same group in 2019, in their retrospective study where only panNETs were evaluated: SUV_max/mean_ failed to predict PFS, whereas the intratumoral textural feature (TF) analysis, assessed by a baseline SSTR-PET, predicted response in terms of PFS [81]. The following studies also did not find any significant relationship between the response to PRRT and SUV_max_ of reference lesions [26] (p 0.12), [73] (p = 0.25), [27] (*p* = 0.59).

**Regarding** ^**18**^**F-FDG PET,** all studies tend to divide patients in PET-positive and PET-negative, depending on a value of SUV_max_ 2.5 or more, as an arbitrary cut off to consider a lesion positive for malignancy (see Table 3). Among eleven papers that analyze ^18^F-FDG PET, only one paper clearly defines that SUV_max_ FDG does not have a predictive role [33], while nine papers show that this criterion could have a predictive role [2, 18, 51, 52, 55, 58, 64, 69, 75]. Finally, one paper reported descriptive data only [53]. As for gallium PET, none of these studies is based on large study populations.

#### “Positive studies”

A retrospective study [69] showed the capacity of FDG PET to characterize the aggressiveness of NETs, both in terms of PFS (*p* = 0.033) and DCR (*p* = 0.02). [64] confirmed these data in a prospective study on pancreatic NETs. Median PFS in the PET-positive group was 21.2 months, while in the PET-negative group, it was 68.7 months (*P* < 0.0002). Median OS was not reached in the PET-negative group and was 63.8 months in the PET-positive group (*p* = 0.006). Similarly, PFS was significantly longer in PET-negative patients in the prospective study from **[**55**]** (*p* = 0.02). Accordingly, DCR was better, but no significantly different, in PET-negative cases (*P* = 0.16). Data from Adnan et al., in their retrospective study which considered only mediastinal NETs, are consistent with what reported thus far, where lower lesional FDG uptake was associated with longer PFS (*p* = 0.002) and OS (*p* = 0.043) [2]. FDG PET predicted survival also in patients with high Ki 67 proliferation index (15–35%) in the retrospective study by [52]: the PFS in FDG negative patients was 65.5 months versus 23.0 months in the FDG-positive patients (no *p* value is reported though).

Finally, [18] did not confirm data about PFS. Median PFS in PET-positive patients was lower but not statistically different from PET-negative patients (*P* = 0.058). However, a significant correlation between a positive 18F-FDG PET and patient death (*P* = 0.03) was found. Moreover, other studies reported the possible role of FDG PET in predicting response to PRRT, with only descriptive data. In particular, a high ^18^F-FDG SUV_max_ is associated with a poor outcome to PRRT and with disease progression [51, 53, 75]. This finding also emerged from a retrospective study [58] conducted on 22 patients with pulmonary NET.

#### “Negative studies”

In a prospective trial on progressive bronchial carcinoids [33] median PFS was lower in FDG PET-positive patients (15.3 months) than in PET-negative patients (26.4 months), but no significant differences were found (*p* = 0.201).

### Tumor burden

Tumor burden (TB) has been evaluated as potential predictor of response to PRRT in sixteen studies conducted from 2003 to 2019. Table 4 shows all selected original articles where TB was evaluated as a predictive factor of response to PRRT. Overall, 1496 patients (807 males and 689 females) with NET were evaluated. One study is a prospective non-randomized phase II clinical trial, two papers are phase I dose escalation studies, while thirteen studies are retrospective. 177Lu was employed in nine studies, 90Y in two and both in five. The main parameter assessed as predictor of response to PRRT or associated with survival was the liver TB, however, authors considered even the presence of other metastatic sites, such as bone metastases, or resection of primary tumor. When liver TB was evaluated, different cut-off were applied, more frequently <25 or < 50% of liver involvement. Several studies did not identify a precise cut-off of liver involvement to stratify patients. The patient populations were affected by neuroendocrine neoplasia (NENs) of the GEP tract in the majority of cases. Only four studies were performed on bronchial NENs or on mixed case series of GEP and thoracic NENs. Almost all patients treated with PRRT were affected by a metastatic and mainly progressive NET. Tumor burden was assessed by CT or MRI in all studies except one and response to treatment was evaluated according to the RECIST criteria in most studies. Five out of sixteen studies consider also the SWOG criteria and only one study evaluated tumor response to treatment by the WHO criteria.

Among sixteen papers that analyze liver TB, four papers [35, 59, 62, 63] did not find any significant result that attributes a predictive role to liver TB, one paper [12] describes the presence of liver metastases as a predictor of overall survival, and eleven identified liver TB as a predictor of response to therapy [6, 14, 18, 23, 24, 43, 46, 49, 50, 77, 82]. Both negative and positive studies comprise retrospective and prospective experiences and no particular features characterize a group relative to the other, nor regarding patient populations or radionuclide employed (177Lu or 90Y).

For instance, some article evaluated the impact of liver TB on ORR after PRRT. [43] observed that after PRRT DCR was achieved in 6 (54.5%) out of 11 patients with diffuse liver metastases and in 21 (91.3%) out of 23 patients without diffuse liver metastases (*p* = 0.01). Similarly, [82] observed that PR and SD at 1 year after PRRT were more frequent in patients with liver TB ≤50% (70.8%) with respect to patients with liver tumor load >50% (38.6%; *p* < 0.001). Authors concluded that hepatic TB is a strong predictor of response to PRRT, of PFS and OS. However, two other studies [23, 63] failed to demonstrate a higher DCR in patients with a low liver TB. Additional reports evaluated the impact of liver TB on disease free survival (DFS) after PRRT. Most of these studies [18, 24, 43, 46, 62, 63, 82] reported in patients with low liver TB (<25% or < 50%, according to the different criteria considered to define the liver tumor load in each study) a statistically significant longer DFS, which ranged from 21 to 49 months after PRRT, relative to patients with a high liver TB where DFS was shorter (ranging from 8 to 28 months). A similar difference was also noted when comparing patients without and with bone metastases: OS was N.R. vs. 25.0 months (*p* = 0.03) and PFS was N.R. vs. 12.0 months (*p* = 0.003). Conversely, these findings were not confirmed by other studies [35, 49, 59] that reported a not significant different DFS when patients were stratified according to the liver TB.

### Grade

Grade (G) has been evaluated as potential predictor of response to PRRT in seventeen papers (2 prospective, 14 retrospective, and 1 retrospective/prospective study -German registry) conducted from 2010 to 2019. Another three retrospective studies stratified the population on the percentage of Ki67 antigen expression [1, 15, 52] and one of them distinguished well-differentiate from poorly differentiated [15]. The population comprised 3561 patients with metastatic and almost all progressive NET, mainly gastroenteropancreatic. ^177^Lu was employed in 11 studies, and both ^90^Y and/or ^177^Lu in 9. Number of cycles ranged from 1 to 8 and cumulative dose was usually between 18.5 and 31.6 GBq. Table 4 shows all selected original articles.

**Table 4:**
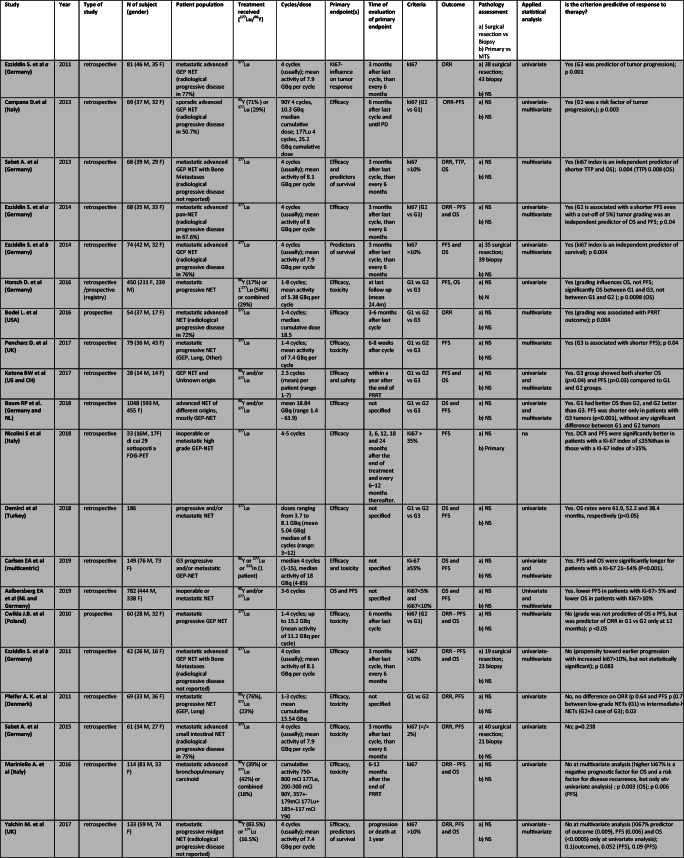
Studies assessing role of *ki67* index as predictor of different outcomes after PRRT. In light gray “Positive studies”, in gray “Negative Studies”

Abbreviations: M, male; F, female; GEP, gastroenteropancreatic; NET, neuroendocrine tumor; GBq, Gigabecquerel; mCi, millicurie; ORR, objective response rate; PFS, progression free survival; OS, overall survival; TTP, time to tumor progression; na, not applicable; ns, not specified

In 20 studies the role of grade in PRRT was analyzed in detail ([14, 17, 21–23, 32, 49, 59, 60] [24], [1, 5, 9, 15, 19, 35, 52, 61, 62, 82]), and this criterion showed an impact on response to PRRT in 14 ([14, 21, 23, 24], Horsch et al 2016, [1, 5, 9, 15, 19, 35, 52, 59, 61]). No association was noted between positive and negative studies and patient populations, sample size, study design or radionuclide employed (^177^Lu or ^90^Y).

### “Positive studies”

In particular six papers defined grade as a predictor of PD [5, 14, 21, 35, 52, 59]. Campana et al. showed that higher grade in NETs is a risk factor for tumor progression after PRRT both at univariate and multivariate analysis (G2 vs G1, p 0.003). [21] reported that ki67 index was higher in PD group than in the other response groups (p 0.001). A rate of 71% of G3 NETs was in PD after PRRT vs 11% in G1 + G2 NETs; p0.001). No statistically difference was demonstrated between G1 and G2 tumors [21]. G3 tumors were also found to be associated with shorter PFS, together with gender, in a paper by [59]. [52] considered for their retrospective analysis only patients with high Ki67 proliferation index (>15%) and found that DCR was 87% in patients with a Ki-67 index of ≤35% (9% PR + 78% SD) and 30% in patients with a Ki-67 index of >35%, although no p is reported. Ten studies reported grade as a predictor of OS [1, 5, 15, 19, 23, 24, 32, 35, 52, 61]. A paper by [24] focalized on the predictors of long-term outcome after PRRT. Among different factors assessed at univariate and multivariate analysis, ki67 index proved to be the strongest predictor of OS. Tumors with ki67 > 10% showed earlier progression after PRRT compared to tumors with Ki67 < 10% (median PFS 19 vs 31 months) and this translated in a shorter survival time (median OS 34 vs 55 months, p 0.004). The same group [23] in a cohort of G1 and G2 panNET found that G2 is associated with a shorter PFS, when analyzed with WHO 2010 cut-off of ki67 2%, and even when the analysis was performed applying a cut-off of 5% (median PFS 24 vs 40 months, p 0.03). Moreover, the study demonstrated that tumor grading was an independent predictor of OS (≤2% vs >2%, median OS not reached vs 49 months respectively). In fact, ki67 index (≤2% or > 2%) remained significant (p 0.044) in the multivariate analysis among factors contributing to OS.

Best OS was achieved, in the large cohort of patients studied by [5], in the G1 group (88 months, *p* = 0.0025), followed by G2. G3 NENs were observed to have the shortest overall survival with 23 months. Compared to G2, OS of G1 patients was significantly longer and significantly shorter in the G3 group (*p* = 0.0023). PFS was found to be significantly shorter only in patients with G3 tumors (*p* < 0.001), without any significant difference between G1 and G2 tumors [5].

Similar results were described by [35], in which study the only significant difference was between the G3 group and the other groups, with the first one showing both shorter OS (*p* = 0.04) and PFS (*p* = 0.03) [35]. [19] also found positive correlation between histological grade and OS rates (*P* < 0.05).

A multi-institutional registry study with prospective follow-up [32], in a large cohort of 450 NET patients, reported that grading had no significant impact on PFS, but determined significant differences in OS between G1 and G3 groups (median OS 33 months for G3 tumors, p 0.0098). However, the statistically significance was not maintained between G1 and G2 groups. In a cohort of only G3 neoplasms (Ki67 > 20%), Carlsen and collaborators defined two different populations on the base of Ki67 index (21–55% and > 55%), finding that median PFS and OS were significantly longer for patients with a Ki-67 21–54% (*P* < 0.001); another parameter evaluated in the study was the grade of differentiation of the primary tumor: well-differentiated tumor had longer OS and PFS than poorly differentiated (P < 0.001) even in an all-G3 neoplasms cohort [15]. Consistent with these results are the findings by [52], also considering only patients with a high Ki67 proliferation index; in this study, a significant difference in PFS (26.3 vs 6.8 months, *p* = 0.005) and OS (52.9 vs 12.6 months, *p* = 0.012) was found using a cut off of a Ki67 of 35%. A different stratification was operated by [1], dividing its large cohort of patients in four quartiles considering the Ki67 valor (Q1 < 2%, Q2 2–5%, Q3 5–10%, Q4 > 10%); in their study they found significantly lower OS for Q4 subgroup only when compared with Q1 subgroup (p = 0.01).

Sabet et al. in 2013 performed a retrospective analysis of a cohort of GEP NET with bone metastases (BM). The results of this paper demonstrated that ki67 > 10% was associated with a shorter TTP (p 0.004). Furthermore, ki67 index was an important prognostic factor that had an impact on OS in this cohort of patients, remaining significant on multivariate analysis (ki67 > 10% vs ki67 < 10% median OS 30 vs 55 months, p 0.008) [61].

A study by Cwikla et al. in 2009 reported that the differences in OS and PFS in patients with G1 and G2 tumors were not statistically significant. On the contrary, G1 and G2 tumors were different in terms of ORR evaluated at 12 months by RECIST criteria (*p* < 0.05) (*Cwikla* et al *2009*).

### “Negative studies”

Six studies [17, 22, 49, 60, 62, 82] reported no significant difference neither in ORR nor in survival (PFS or OS) according to G after PRRT. In some of these studies [49, 82] the influence of G on ORR, PFS and OS was significant only at univariate analysis but not maintained at subsequently multivariate analysis.

## The NETest

The parameters so far described to predict tumor response and patient survival are all based on radiological, istological or nuclear evidences; different from that, the “NETest” consists of a liquid biopsy, evaluating in real time the transcriptional tumor profile (or its “gene signature”) by blood sample. The NETest aims at defining the neoplasm precise biological activity, including diagnostic accuracy, prognostic value, and predictive therapeutic assessment.

The push for the development of such test are to be found in some of the limitations of most prognostic and predictive factors for NENs evaluation, such as low reproducibility and high inter-variability. The diagnostic accuracy of this mRNA-based evaluation seems to be able to identify all NEN types, including small non metastatic tumors.

Regarding the predictive efficacy of the NETest prior to PRRT, an algorithm that integrates specific gene transcripts with tissue Ki67 values (either from primary or metastasis) was developed by the authors to generate a PRRT Predictive Quotient (PPQ) characterized by two prediction outputs: “PRRT-responder” or “PRRT-non-responder”. The authors developed and validated the PPQ in three prospective studies, enrolling a total of 158 ^177^LU-PRRT treated patients: in these different cohorts, fifty-one marker genes were measured to best predict PRRT efficacy and it was observed that PPQ correlated accurately with PRRT both in responders (97%) and in non-responders (91%); even changes in gene expression reflected in treatment response assessment scored with RECIST. Conversely, no gene signature is available at the moment for the assessment of the risk for mielo- or nephrotoxicity in PRRT [10]. They conclude that the NETest showed results thus far unmatched by other commonly used markers.

The impressive results showed in the paper by [10] are undoubtedly promising, still, yet to be confirmed in other studies, which are certainly eagerly needed, in order to further assess the value and accuracy of NETest in diagnosing and predicting outcome in patients with NETs.

## Discussion

PRRT is now a well defined therapeutic option to treat GEP NET patients after failure of SSA, while predictors of tumor response to PRRT and patient survival after treatment has not yet been found.

The present study tried to define the identikit of the “perfect patient” to candidate to PRRT. However, to distinguish between predictors of response factors and prognostic factors is challenging. The inhomogeneous distribution of primaries, small sample size of the studies and different outcomes taken into consideration, together with the different follow up and timing of evaluation, may have hampered the chance to identify which patients may benefit from PRRT more than others in terms of survival or tumor response.

Regarding **primary origin**, GEP NET seem to be more responsive than non-GEP NET, both in terms of ORR [4] than PFS [4, 5], while among the GEP tumors, panNET show a better ORR than small intestinal NET[60].

If the studies reporting primary site to be a predictor factor of response to PRRT are few, little more are the ones showing absence of any correlation between primary tumor and ORR [17, 59, 68, 70, 78, 79]. The reported evidence that panNET respond better than small intestinal NET, but without any significant difference in PFS, is likely to correlate to the known phenomenon of shorter PFS of patients with panNET despite a more pronounced initial response to PRRT in terms of ORR [44, 46]. In the setting of NEN with unknown primary, PRRT is a potentially effective therapeutic option, although there are not univocal data in literature [3].

Although also concerning **tumor burden** (TB) no concordant conclusions can be drawn, most of these studies ([18, 23, 24, 46] e [43, 77, 82], [49], Kolasinska-Cwilla et al. ) reported, in patients with low liver TB (<25% or < 50%, according to the different criteria considered to define the liver tumor load in each study), a statistically significant longer DFS after PRRT than in patients with a high liver TB (DFS range 21–49 months vs 8–28 months). Not only the presence of liver metastases, but also the overall tumor load, including other metastatic sites and even the primary tumor (if not resectable), should be taken into account when considering PRRT as appropriate choice for patients with advanced NETs. For instance, some evidences reported that patients with bone metastases had a higher risk of progression after PRRT than those without bone metastases [46]. Similarly, the stage of disease seems to significantly affect the hazard ratio for disease progression after PRRT [14]. In summary, some evidences from retrospective studies suggest that patients with a low TB, especially in the liver, may benefit most from PRRT. Therefore, waiting for tumor progression before PRRT administration or choosing PRRT for patients with a large tumor load might be not appropriate. The TB must be included and carefully weighed in the multidisciplinary discussion of the individual patient for the indication to PRRT.

**Somatostatin receptor imaging** is a mandatory prerequisite for the use of PRRT. First studies were based on Somatostatin Receptor Scintigraphy (SRS) with 111In-pentetreotide (Octreoscan) but, in recent years, 68Ga-DOTA-peptides PET/CT showed a better diagnostic performance than SRS. At now, 68Ga-PET represents the method of choice for the “in vivo” evaluation of SSTR expression, allowing also the calculation of semiquantitative parameters such as SUVmax and improving imaging resolution. At this purpose, the collection of further data with the larger number of PET scans performed today, will allow us to drawn conclusion in a comparative, retrospective analysis including the Octreoscan era. Among the papers analyzed, a half identified SUVmax as a predictor of response to therapy, whereas the remaining half either did not find significance or identified other parameters able to predict outcome of PRRT treatment. Three studies identified a SUVmax cut-off to select patients for PRRT, but to establish the potential ability to predict response to PRRT further studies are needed. In conclusion, Gallium uptake is an inclusion criterion for PRRT, but poorly correlates with response to therapy and it is not a predictive factor in an individual patient. This confirms that the expression of sstr is not the unique determinant of efficacy of PRRT [24].

On the contrary, ^**18**^**F-FDG PET** seems to have a role in predicting disease progression, tumor response and survival in patients with advanced NETs, treated with PRRT. Most studies suggest that the patients with negative baseline scan may benefit from PRRT more than positive patients, also showing that a high 18F-FDG SUV_max_ is associated with a poor outcome to PRRT and with disease progression [51–53, 75]. Similar results also emerged in 22 patients with pulmonary NET, evaluated retrospectively [58]. Therefore, ^**18**^**F-FDG PET** must be taken into account during therapeutic decision-making and multidisciplinary assessment of different patients.

The most interesting and promising criterion to predict response to PRRT seems to be **ki67 index**. Although in general the ki67 index is being recognized as a powerful determinant of survival in patients with GEP NETs and it is known as a major prognostic factor for NETs [56, 57, 65], its relevance in metastatic disease and potential cutoff values for the different treatment modalities are still undefined because of a lack of data. Keeping this concept in mind, nevertheless we have found many evidences in the literature that attribute to the degree a predictive value of response (ORR) or survival (PFS) after PRRT [1, 5, 9, 14, 15, 19, 21, 23, 24, 32, 35, 52, 59, 61].

ki67 index proved to be the strongest predictor of outcome in that patient cohort [24]. Authors reported that, even though G2 tumors with a ki67 index >10% respond in a similar manner to lesions with ki67 < 10% (accordingly to their previous results above stated), G2 NENs with ki67 > 10% show earlier progression after PRRT (median PFS of 19 vs 31 months). Although it is well known that grade affect prognoses of NENs in general, this evidence shows that grade provides prognostic stratification in a uniformly treated cohort of PRRT pretreated patients.

Moreover, [14] confirmed the role of grade as crucial therapeutic prognostic factors for response to PRRT in NETs. In fact, grade was a risk factor for PD at multivariate analysis (NET G2 vs NET G1, HR 3.481, *p* = 0.003). Similar findings were reported in another five recent studies [1, 5, 15, 35], where different populations were analyzed and different Ki67 proliferation index thresholds used, but all found significance about longer survival associated with lower grade.

Again, the recent study by [9] demonstrated that multiple regression analysis identified only grading as factors associated with PRRT outcome (p 0.004), a part from the new promising NET test.

Proliferation index calculated by ki67 labeling has some limitation that could influence part of these reported results. One of these limits is the intratumoral heterogeneity of ki67 index [28]. Although it is well known that Ki67 may differ between primary lesion to synchronous or metachronous metastases and even between two different sites of metastases, [28] in none, but one, of cited papers it is specified whether the pathology sample was harvested from the primary or metastatic lesions. The origin from surgical specimen or simple biopsy was reported only in the 25% of the considered report, as well. Anyway, despite such limitation and potentially confounding factors, evidence about the predictive role of grade on PRRT was found by most of the authors. In this respect, to overcome issues of temporal and spatial inaccuracy of ki67 index, an alternative tool such as a FDG PET could provide a whole imaging of the aggressiveness of tumor, with a picture of all metastatic sites together and at same time ([7], Garin et al 2009).

However, we must to consider that other studies ([22, 49,60, 62, 82]*)* in literature do not define grade as predictor of response after PRRT and one study reported some conflicting, but finally negative, results [17]. Another limit of these conclusions about grade is that some authors in their papers consider grade cathegory (WHO 2010, G1, G2, G3 groups), while others consider different cut-off of ki67 such as 2%, 5%, 10% and 20%. A cut-off of ki67 has not been univocally identified.

Finally, we must consider that PRRT efficacy is affected by previous therapies and little evidence is available about the appropriate position of PRRT in the NET treatment sequence. In this respect it would have been of great interest to assess the response to PRRT based on the positioning of this therapy: in first, second line or further lines. However, of course, this data is not reported in all studies in a homogeneous way. Two of the wider and most recent case series have only partially analyzed the problem [1, 5].

Baum et al., out of a study population of more than 1000 patients, showed a significant disadvantage in terms of OS in a subgroup of patients who had received PRRT after more than three previous lines, and who represented around 19% of the enrolled population. In contrast, statistically longer OS was recorded in the group of second-line PRRT-treated patients (28% of patients enrolled).

This data, however, can also be interpreted as a consequence of the fact that OS is a factor that reflects patient’s prognosis and it is evident that a patient, who has already performed more than 3 treatment lines, is later in his history natural disease. The outcome of these patients will be probably more influenced by their advanced stage than sequence of therapies received. In the series published in 2019 from Aalbersberg et al., while reporting the percentages of treatment naive patients, of those in the first, second or subsequent lines, then does not analyze the response based on treatment line. On the other hand, a lower effectiveness of PRRT when performed after chemotherapy or interferon is reported. The NETTER-1 trial [74] demonstrated a lower risk of disease progression or death of 79%, in a setting of second line treatment, after failure of “cold” somatostatin analogues. For the future, further insight on sequence will be derived from the NETTER-2 trial, although it will be conduct on a different population (*clinicaltrials.gov*).

Amongst future prospective, the recent study by [10] demonstrated that an algorithm including circulating NET transcripts and Ki67 proliferation index from primary or metastatic lesions correlated accurately with PRRT responders vs non responders and predicted PRRT efficacy. The gene signature that characterize the **NETest** showed promising and impressive results on discerning such patients, but not enough studies have confirmed these results so far and NETest, to date, is not routinely performed.

In conclusion, to date we have mostly prognostic (tumor burden, FDG uptake, grade) than predictive factors to predict efficacy for PRRT. The perfect patient, selected by Gallium DOTA-peptide PET uptake (or other somatostatin receptor imaging), will be likely characterized by a FDG PET negative scans, a relatively limited liver TB, a ki67 index <20% and will respond to PRRT irrespective to primary tumor origin. Nevertheless, at this moment the identikit of the perfect patient for PRRT therapy is a puzzle without some pieces. Still we cannot disregard a multidisciplinary discussion of the individual case to select the patients who will mostly benefit from the PRRT treatment.

## References

[1] Aalbersberg EA, Huizing DMV, Walraven I, de Wit-van der Veen BJ, Kulkarni HR, Singh A, Stokkel MPM, Baum RP (2019). Parameters to predict progression-free and overall survival after peptide receptor radionuclide therapy: a multivariate analysis in 782 patients. J Nucl Med.

[2] Adnan A, Kudachi S, Ramesh S, Prabhash K, Basu S (2019). Metastatic or locally advanced mediastinal neuroendocrine tumours: outcome with 177Lu-DOTATATE-based peptide receptor radionuclide therapy and assessment of prognostic factors. Nucl Med Commun.

[3] Alexandraki (2019). Current Concepts in the Diagnosis and Management of Neuroendocrine Neoplasms of Unknown Primary Origin. Minerva Endocrinol..

[4] Baum RP, Kluge AW, Kulkarni H, Schorr-Neufing U, Niepsch K, Bitterlich N, van Echteld CJ (2016). [(177)Lu-DOTA](0)-D-Phe(1)-Tyr(3)-Octreotide ((177)Lu-DOTATOC) for peptide receptor radiotherapy in patients with advanced neuroendocrine Tumours: a phase-II study. Theranostics.

[5] Baum RP, Kulkarni HR, Singh A, Kaemmerer D, Mueller D, Prasad V, Hommann M, Robiller FC, Niepsch K, Franz H, Jochems A, Lambin P, Hörsch D (2018). Results and adverse events of personalized peptide receptor radionuclide therapy with 90Yttrium and 177Lutetium in 1048 patients with neuroendocrine neoplasms. Oncotarget.

[6] Bertani E, Fazio N, Radice D, Zardini C, Grana C, Bodei L, Funicelli L, Ferrari C, Spada F, Partelli S, Falconi M (2016). Resection of the primary tumor followed by peptide receptor radionuclide therapy as upfront strategy for the treatment of G1-G2 pancreatic neuroendocrine tumors with Unresectable liver metastases. Ann Surg Oncol.

[7] Binderup T, Knigge U, Loft A, Federspiel B, Kjaer A (2010). 18F-fluorodeoxyglucose positron emission tomography predicts survival of patients with neuroendocrine tumors. Clin Cancer Res.

[8] Bodei L, Cremonesi M, Grana CM, Fazio N, Iodice S, Baio SM, Bartolomei M, Lombardo D, Ferrari ME, Sansovini M, Chinol M, Paganelli G (2011). Peptide receptor radionuclide therapy with ^177^Lu-DOTATATE: the IEO phase I-II study. Eur J Nucl Med Mol Imaging.

[9] Bodei L, Kidd M, Modlin IM, Severi S, Drozdov I, Nicolini S, Kwekkeboom DJ, Krenning EP, Baum RP, Paganelli G (2016). Measurement of circulating transcripts and gene cluster analysis predicts and defines therapeutic efficacy of peptide receptor radionuclide therapy (PRRT) in neuroendocrine tumors. Eur J Nucl Med Mol Imaging.

[10] Bodei L, Kidd MS, Singh A, van der Zwan WA, Severi S, Drozdov IA, Cwikla J, Baum RP, Kwekkeboom DJ, Paganelli G, Krenning EP, Modlin IM (2018). PRRT genomic signature in blood for prediction of 177Lu-octreotate efficacy. Eur J Nucl Med Mol Imaging.

[11] Borson-Chazot F, Anthony L, Benson AB, Oberg K, Grossman AB, Connolly M, Bouterfa H, Li Y, Kacena KA, LaFrance N, Pauwels SA (2010). 90Y-edotreotide for metastatic carcinoid refractory to octreotide. J Clin Oncol.

[12] Brabander T, van der Zwan WA, Teunissen JJM, Kam BLR, Feelders RA, de Herder WW, van Eijck CHJ, Franssen GJH, Krenning EP, Kwekkeboom DJ (2017). Long-Term Efficacy, Survival, and Safety of [177Lu-DOTA0,Tyr3]octreotate in Patients with Gastroenteropancreatic and Bronchial Neuroendocrine Tumors. Clin Cancer Res.

[13] Bushnell DL, O'Dorisio TM, O'Dorisio MS, Menda Y, Hicks RJ, Van Cutsem E, Baulieu JL, Borson-Chazot F, Anthony L, Benson AB, Oberg K, Grossman AB, Connolly M, Bouterfa H, Li Y, Kacena KA, LaFrance N, Pauwels SA (2010). 90Y-edotreotide for metastatic carcinoid refractory to octreotide. J Clin Oncol.

[14] Campana D, Capurso G, Partelli S, Nori F, Panzuto F, Tamburrino D, Cacciari G, Delle Fave G, Falconi M, Tomassetti P (2013). Radiolabelled somatostatin analogue treatment in gastroenteropancreatic neuroendocrine tumours: factors associated with response and suggestions for therapeutic sequence. Eur J Nucl Med Mol Imaging.

[15] Carlsen EA, Fazio N, Granberg D, Grozinsky-Glasberg S, Ahmadzadehfar H, Grana CM, Zandee WT, Cwikla J, Walter MA, Oturai PS, Rinke A, Weaver A, Frilling A, Gritti S, Arveschoug AK, Meirovitz A, Knigge U, Sorbye H (2019). Peptide receptor radionuclide therapy in gastroenteropancreatic NEN G3: a multicenter cohort study. Endocr Relat Cancer.

[16] clinicaltrials.gov. Available from: https://clinicaltrials.gov/.

[17] Cwikla JB, Sankowski A, Seklecka N, Buscombe JR, Nasierowska-Guttmejer A, Jeziorski KG, Mikolajczak R, Pawlak D, Stepien K, Walecki J (2010). Efficacy of radionuclide treatment DOTATATE Y-90 in patients with progressive metastatic gastroenteropancreatic neuroendocrine carcinomas (GEP-NETs): a phase II study. Ann Oncol.

[18] Delpassand ES, Samarghandi A, Zamanian S, Wolin EM, Hamiditabar M, Espenan GD, Erion JL, O'Dorisio TM, Kvols LK, Simon J, Wolfangel R, Camp A, Krenning EP, Mojtahedi A (2014). Peptide receptor radionuclide therapy with 177Lu-DOTATATE for patients with somatostatin receptor-expressing neuroendocrine tumors: the first US phase 2 experience. Pancreas.

[19] Demirci E, Kabasakal L, Toklu T, Ocak M, Şahin OE, Alan-Selcuk N, Araman A (2018). 177Lu-DOTATATE therapy in patients with neuroendocrine tumours including high-grade (WHO G3) neuroendocrine tumours: response to treatment and long-term survival update. Nucl Med Commun.

[20] Dumont RA, Seiler D, Marincek N, Brunner P, Radojewski P, Rochlitz C, Müller-Brand J, Maecke HR, Briel M, Walter MA (2014). Survival after somatostatin based radiopeptide therapy with (90)Y-DOTATOC vs. (90)Y-DOTATOC plus (177)Lu-DOTATOC in metastasized gastrinoma. Am J Nucl Med Mol Imaging.

[21] Ezziddin S, Opitz M, Attassi M, Biermann K, Sabet A, Guhlke S, Brockmann H, Willinek W, Wardelmann E, Biersack HJ, Ahmadzadehfar H (2011). Impact of the Ki-67 proliferation index on response to peptide receptor radionuclide therapy. Eur J Nucl Med Mol Imaging.

[22] Ezziddin S, Sabet A, Heinemann F, Yong-Hing CJ, Ahmadzadehfar H, Guhlke S, Höller T, Willinek W, Boy C, Biersack HJ (2011). Response and long-term control of bone metastases after peptide receptor radionuclide therapy with (177)Lu-octreotate. J Nucl Med..

[23] Ezziddin S, Khalaf F, Vanezi M, Haslerud T, Mayer K, Al Zreiqat A, Willinek W, Biersack HJ, Sabet A (2014). Outcome of peptide receptor radionuclide therapy with 177Lu-octreotate in advanced grade 1/2 pancreatic neuroendocrine tumours. Eur J Nucl Med Mol Imaging..

[24] Ezziddin S, Attassi M, Yong-Hing CJ, Ahmadzadehfar H, Willinek W, Grünwald F, Guhlke S, Biersack HJ, Sabet A (2014). Predictors of long-term outcome in patients with well-differentiated gastroenteropancreatic neuroendocrine tumors after peptide receptor radionuclide therapy with 177Lu-octreotate. J Nucl Med..

[25] Filice A, Fraternali A, Frasoldati A, Asti M, Grassi E, Massi L, Sollini M, Froio A, Erba PA, Versari A (2012). Radiolabeled somatostatin analogues therapy in advanced neuroendocrine tumors: a single Centre experience. J Oncol.

[26] Gabriel M, Oberauer A, Dobrozemsky G, Decristoforo C, Putzer D, Kendler D, Uprimny C, Kovacs P, Bale R, Virgolini IJ (2009). 68Ga-DOTA-Tyr3-octreotide PET for assessing response to somatostatin-receptor-mediated radionuclide therapy. J Nucl Med.

[27] Gabriel M, Nilica B, Kaiser B, Virgolini IJ (2019). Twelve-year follow-up after peptide receptor radionuclide therapy. J Nucl Med.

[28] Grillo F, Albertelli M, Brisigotti MP, Borra T, Boschetti M, Fiocca R, Ferone D, Mastracci L (2016). Grade increases in Gastroenteropancreatic neuroendocrine tumor metastases compared to the primary tumor. Neuroendocrinology.

[29] Grozinsky-Glasberg S, Barak D, Fraenkel M, Walter MA, Müeller-Brand J, Eckstein J, Applebaum L, Shimon I, Gross DJ (2011). Peptide receptor radioligand therapy is an effective treatment for the long-term stabilization of malignant gastrinomas. Cancer..

[30] Hamiditabar M, Ali M, Roys J, Wolin EM, TM OD, Ranganathan D, Tworowska I, Strosberg JR, Delpassand ES (2017). Peptide receptor radionuclide therapy with 177Lu-Octreotate in patients with Somatostatin receptor expressing neuroendocrine tumors: six Years' assessment. Clin Nucl Med.

[31] Haug AR, Auernhammer CJ, Wängler B, Schmidt GP, Uebleis C, Göke B, Cumming P, Bartenstein P, Tiling R, Hacker M (2010). 68Ga-DOTATATE PET/CT for the early prediction of response to somatostatin receptor-mediated radionuclide therapy in patients with well-differentiated neuroendocrine tumors. J Nucl Med.

[32] Hörsch D, Ezziddin S, Haug A, Gratz KF, Dunkelmann S, Miederer M, Schreckenberger M (2016). Krause BJ5, Bengel FM4, Bartenstein P3, Biersack HJ7, Pöpperl G8, Baum RP. Effectiveness and side-effects of peptide receptor radionuclide therapy for neuroendocrine neoplasms in Germany: a multi-institutional registry study with prospective follow-up. Eur J Cancer.

[33] Ianniello A, Sansovini M, Severi S, Nicolini S, Grana CM, Massri K, Bongiovanni A, Antonuzzo L, Di Iorio V, Sarnelli A, Caroli P, Monti M, Scarpi E, Paganelli G (2016). Peptide receptor radionuclide therapy with (177)Lu-DOTATATE in advanced bronchial carcinoids: prognostic role of thyroid transcription factor 1 and (18)F-FDG PET. Eur J Nucl Med Mol Imaging.

[34] Imhof A, Brunner P, Marincek N, Briel M, Schindler C, Rasch H, Mäcke HR, Rochlitz C, Müller-Brand J, Walter MA (2011). Response, survival, and long-term toxicity after therapy with the radiolabeled somatostatin analogue [90Y-DOTA]-TOC in metastasized neuroendocrine cancers. J Clin Oncol.

[35] Katona BW, Roccaro GA, Soulen MC, Yang YX, Bennett BJ, Riff BP, Glynn RA, Wild D, Nicolas GP, Pryma DA, Teitelbaum UR, Metz DC (2017). Efficacy of peptide receptor radionuclide therapy in a United States-based cohort of metastatic neuroendocrine tumor patients: single-institution retrospective analysis. Pancreas.

[36] Khan S, Krenning EP, van Essen M, Kam BL, Teunissen JJ, Kwekkeboom DJ (2011). Quality of life in 265 patients with gastroenteropancreatic or bronchial neuroendocrine tumors treated with [177Lu-DOTA0,Tyr3]octreotate. J Nucl Med.

[37] Knapp WH, Arnold R. Consensus protocol of the German Society of Nuclear Medicine and the section NET of the German Society of Endocrinology; workshop peptide receptor radionuclide therapy. Updated 2 December 2008. http://www.netregister.org/wDeutsch/ne_tumore/therapiemoeglichkeiten/peptid_radiorezeptor_therapie.php

[38] Kratochwil C, Stefanova M, Mavriopoulou E, Holland-Letz T, Dimitrakopoulou-Strauss A, Afshar-Oromieh A, Mier W, Haberkorn U, Giesel FL (2015). SUV of [68Ga]DOTATOC-PET/CT predicts response probability of PRRT in neuroendocrine tumors. Mol Imaging Biol.

[39] Krenning EP, Kwekkeboom DJ, Bakker WH, Breeman WA, Kooij PP, Oei HY, van Hagen M, Postema PT, de Jong M, Reubi JC (1993). Somatostatin receptor scintigraphy with [111In-DTPA-D-Phe1]- and [123I-Tyr3]-octreotide: the Rotterdam experience with more than 1000 patients. Eur J Nucl Med.

[40] Krenning EP, Kooij PP, Bakker WH, Breeman WA, Postema PT, Kwekkeboom DJ, Oei HY, de Jong M, Visser TJ, Reijs AE (1994). Radiotherapy with a radiolabeled somatostatin analogue, [111In-DTPA-D-Phe1]-octreotide. A case history. Ann N Y Acad Sci.

[41] Krenning EP, de Jong M, Kooij PP, Breeman WA, Bakker WH, de Herder WW, van Eijck CH, Kwekkeboom DJ, Jamar F, Pauwels S, Valkema R (1999). Radiolabelled somatostatin analogue(s) for peptide receptor scintigraphy and radionuclide therapy. Ann Oncol.

[42] Kunikowska J, Pawlak D, Bąk MI, Kos-Kudła B, Mikołajczak R, Królicki L (2017). Long-term results and tolerability of tandem peptide receptor radionuclide therapy with 90Y/177Lu-DOTATATE in neuroendocrine tumors with respect to the primary location: a 10-year study. Ann Nucl Med.

[43] Kwekkeboom DJ, Bakker WH, Kam BL, Teunissen JJM, Kooij PPM, De Herder WW, Erion JL (2003). Treatment of patients with gastro-entero-pancreatic (GEP) tumours with the novel radiolabelled somatostatin analogue [177Lu-DOTA0, Tyr3] octreotate. Eur J Nucl Med Mol Imaging.

[44] Kwekkeboom DJ, Mueller-Brand J, Paganelli G, Anthony LB, Pauwels S, Kvols LK, et al. Overview of results of peptide receptor radionuclide therapy with 3 radiolabeled somatostatin analogs. J Nucl Med. 2005a;46(Suppl 1):62S–6S. 15653653

[45] Kwekkeboom DJ, Teunissen JJ, Bakker WH, Kooij PP, de Herder WW, Feelders RA, van Eijck CH, Esser JP, Kam BL, Krenning EP (2005). Radiolabeled somatostatin analog [177Lu-DOTA0,Tyr3]octreotate in patients with endocrine gastroenteropancreatic tumors. J Clin Oncol..

[46] Kwekkeboom DJ, de Herder WW, Kam BL, van Eijck CH, van Essen M, Kooij PP, Feelders RA, van Aken MO, Krenning EP (2008). Treatment with the radiolabeled somatostatin analog [177 Lu-DOTA 0,Tyr3]octreotate: toxicity, efficacy, and survival. J Clin Oncol.

[47] Kwekkeboom DJ, Krenning EP (2016). Peptide receptor radionuclide therapy in the treatment of neuroendocrine tumors. Hematol Oncol Clin North Am.

[48] Makis W, McCann K, McEwan AJ (2015). The challenges of treating Paraganglioma patients with (177)Lu-DOTATATE PRRT: catecholamine crises, tumor Lysis syndrome and the need for modification of treatment protocols. Nucl Med Mol Imaging.

[49] Mariniello A, Bodei L, Tinelli C, Baio SM, Gilardi L, Colandrea M, Papi S, Valmadre G, Fazio N, Galetta D, Paganelli G, Grana CM (2016). Long-term results of PRRT in advanced bronchopulmonary carcinoid. Eur J Nucl Med Mol Imaging.

[50] Marincek N, Jörg AC, Brunner P, Schindler C, Koller MT, Rochlitz C, Müller-Brand J, Maecke HR, Briel M, Walter MA (2013). Somatostatin-based radiotherapy with [90Y-DOTA]-TOC in neuroendocrine tumors: long-term outcome of a phase I dose escalation study. J Transl Med.

[51] Nilica B, Waitz D, Stevanovic V, Uprimny C, Kendler D, Buxbaum S, Warwitz B, Gerardo L, Henninger B, Virgolini I, Rodrigues M (2016). Direct comparison of (68)Ga-DOTA-TOC and (18)F-FDG PET/CT in the follow-up of patients with neuroendocrine tumour treated with the first full peptide receptor radionuclide therapy cycle. Eur J Nucl Med Mol Imaging.

[52] Nicolini S, Severi S, Ianniello A, Sansovini M, Ambrosetti A, Bongiovanni A, Scarpi E, Di Mauro F, Rossi A, Matteucci F, Paganelli G (2018). Investigation of receptor radionuclide therapy with 177Lu-DOTATATE in patients with GEP-NEN and a high Ki-67 proliferation index. Eur J Nucl Med Mol Imaging.

[53] Oh S, Prasad V, Lee DS, Baum RP. Effect of peptide receptor radionuclide therapy on Somatostatin receptor status and glucose metabolism in neuroendocrine tumors: Intraindividual comparison of Ga-68 DOTANOC PET/CT and F-18 FDG PET/CT. Int J Mol Imaging 2011;2011:524130. doi: 10.1155/2011/524130. 10.1155/2011/524130 PMC3216394 22121482

[54] Öksüz MÖ, Winter L, Pfannenberg C, Reischl G, Müssig K, Bares R, Dittmann H (2014). Peptide receptor radionuclide therapy of neuroendocrine tumors with (90)Y-DOTATOC: is treatment response predictable by pre-therapeutic uptake of (68)Ga-DOTATOC?. Diagn Interv Imaging.

[55] Paganelli G, Sansovini M, Ambrosetti A, Severi S, Monti M, Scarpi E, Donati C, Ianniello A, Matteucci F, Amadori D (2014). 177 Lu-Dota-octreotate radionuclide therapy of advanced gastrointestinal neuroendocrine tumors: results from a phase II study. Eur J Nucl Med Mol Imaging.

[56] Pape UF, Jann H, Müller-Nordhorn J, Bockelbrink A, Berndt U, Willich SN, Koch M, Röcken C, Rindi G, Wiedenmann B (2008). Prognostic relevance of a novel TNM classification system for upper gastroenteropancreatic neuroendocrine tumors. Cancer.

[57] Pape UF, Berndt U, Müller-Nordhorn J, Böhmig M, Roll S, Koch M, Willich SN, Wiedenmann B (2008). Prognostic factors of long-term outcome in gastroenteropancreatic neuroendocrine tumours. Endocr Relat Cancer.

[58] Parghane RV, Talole S, Prabhash K, Basu S (2017). Clinical response profile of metastatic/advanced pulmonary neuroendocrine tumors to peptide receptor radionuclide therapy with 177Lu-DOTATATE. Clin Nucl Med.

[59] Pencharz D, Walker M, Yalchin M, Quigley AM, Caplin M, Toumpanakis C (2017). Navalkissoor S early efficacy of and toxicity from lutetium-177-DOTATATE treatment in patients with progressive metastatic NET. Nucl Med Commun.

[60] Pfeifer AK, Gregersen T, Grønbæk H, Hansen CP, Müller-Brand J, Herskind Bruun K, Krogh K, Kjær A, Knigge U (2011). Peptide receptor radionuclide therapy with Y-DOTATOC and (177)Lu-DOTATOC in advanced neuroendocrine tumors: results from a Danish cohort treated in Switzerland. Neuroendocrinology..

[61] Sabet A, Khalaf F, Haslerud T, Al-Zreiqat A, Sabet A, Simon B, Pöppel TD, Biersack HJ, Ezziddin S (2013). Bone metastases in GEP-NET: response and long-term outcome after PRRT from a follow-up analysis. Am J Nucl Med Mol Imaging..

[62] Sabet A, Dautzenberg K, Haslerud T, Aouf A, Sabet A, Simon B, Ezziddin S (2015). Specific efficacy of peptide receptor radionuclide therapy with 177Lu-octreotate in advanced neuroendocrine tumours of the small intestine. Eur J Nucl Med Mol Imaging.

[63] Sabet A, Haug AR, Eiden C, Auernhammer CJ, Simon B (2017). Bartenstein, P, Ezziddin, S. efficacy of peptide receptor radionuclide therapy with 177Lu-octreotate in metastatic pulmonary neuroendocrine tumors: a dual-Centre analysis. Am J Nucl Med Mol Imaging.

[64] Sansovini M, Severi S, Ianniello A, Nicolini S, Fantini L, Mezzenga E, Ferroni F, Scarpi E, Monti M, Bongiovanni A, Cingarlini S, Grana CM, Bodei L, Paganelli G (2017). Long-term follow-up and role of FDG PET in advanced pancreatic neuroendocrine patients treated with 177Lu-D OTATATE. Eur J Nucl Med Mol Imaging.

[65] Scarpa A, Mantovani W, Capelli P, Beghelli S, Boninsegna L, Bettini R, Panzuto F, Pederzoli P, Delle Fave G, Falconi M (2010). Pancreatic endocrine tumors: improved TNM staging and histopathological grading permit a clinically efficient prognostic stratification of patients. Mod Pathol.

[66] Scheidhauer K, Miederer M, Gaertner FC (2009). PET-CT for neuroendocrine tumors and nuclear medicine therapy options. [Article in German]. Radiologe.

[67] Seregni E, Maccauro M, Coliva A, Castellani MR, Bajetta E, Aliberti G, Vellani C, Chiesa C, Martinetti A, Bogni A, Bombardieri E (2010). Treatment with tandem [(90)Y]DOTA-TATE and [(177)Lu] DOTA-TATE of neuroendocrine tumors refractory to conventional therapy: preliminary results. Q J Nucl Med Mol Imaging.

[68] Seregni E, Maccauro M, Chiesa C, Mariani L, Pascali C, Mazzaferro V, De Braud F, Buzzoni R, Milione M, Lorenzoni A, Bogni A, Coliva A, Lo Vullo S, Bombardieri E (2014). Treatment with tandem [90Y]DOTA-TATE and [177Lu]DOTA-TATE of neuroendocrine tumours refractory to conventional therapy. Eur J Nucl Med Mol Imaging.

[69] Severi S, Nanni O, Bodei L, Sansovini M, Ianniello A, Nicoletti S, Scarpi E, Matteucci F, Gilardi L, Paganelli G (2013). Role of 18FDG PET/CT in patients treated with 177Lu-DOTATATE for advanced differentiated neuroendocrine tumours. Eur J Nucl Med Mol Imaging.

[70] Sharma N, Naraev BG, Engelman EG, Zimmerman MB, Bushnell DL, TM OD, MS OD, Menda Y, Müller-Brand J, Howe JR, Halfdanarson TR (2017). Peptide receptor radionuclide therapy outcomes in a north American cohort with metastatic well-differentiated neuroendocrine tumors. Pancreas..

[71] Sharma R, Wang WM, Yusuf S, Evans J, Ramaswami R, Wernig F, Frilling A, Mauri F, Al-Nahhas A, Aboagye EO, Barwick TD (2019). 68Ga-DOTATATE PET/CT parameters predict response to peptide receptor radionuclide therapy in neuroendocrine tumours. Radiother Oncol.

[72] Sowa-Staszczak A, Pach D, Stefańska A, Tomaszuk M, Lenda-Tracz W, Mikołajczak R, Pawlak D, Chrzan R, Gilis-Januszewska A, Przybylik-Mazurek E, Hubalewska-Dydejczyk A (2011). Can treatment using radiolabelled somatostatin analogue increase the survival rate in patients with non-functioning neuroendocrine pancreatic tumours?. Nucl Med Rev Cent East Eur.

[73] Soydal Ç, Peker A, Özkan E, Küçük ÖN, Kir MK (2016). The role of baseline Ga-68 DOTATATE positron emission tomography/computed tomography in the prediction of response to fixed-dose peptide receptor radionuclide therapy with Lu-177 DOTATATE. Turk J Med Sci.

[74] Strosberg J, El-Haddad G, Wolin E, Hendifar A, Yao J, Chasen B, Mittra E, Kunz PL, Kulke MH, Jacene H, Bushnell D, O'Dorisio TM, Baum RP, Kulkarni HR, Caplin M, Lebtahi R, Hobday T, Delpassand E, Van Cutsem E, Benson A, Srirajaskanthan R, Pavel M, Mora J, Berlin J, Grande E, Reed N, Seregni E, Öberg K, Lopera Sierra M, Santoro P, Thevenet T, Erion JL, Ruszniewski P, Kwekkeboom D, Krenning E (2017). NETTER-1 Trial Investigators. Phase 3 Trial of 177Lu-Dotatate for Midgut Neuroendocrine Tumors. N Engl J Med..

[75] Thapa P, Ranade R, Ostwal V, Shrikhande SV, Goel M, Basu S (2016). Performance of 177Lu-DOTATATE-based peptide receptor radionuclide therapy in metastatic gastroenteropancreatic neuroendocrine tumor: a multiparametric response evaluation correlating with primary tumor site, tumor proliferation index, and dual tracer imaging characteristics. Nucl Med Commun.

[76] Vaisman F, Rosado de Castro PH, Lopes FP, Kendler DB, Pessoa CH, Bulzico DA, de Carvalho Leal D, Vilhena B, Vaisman M, Carneiro M, Corbo R (2015). Is there a role for peptide receptor radionuclide therapy in medullary thyroid cancer?. Clin Nucl Med.

[77] Valkema R, Pauwels S, Kvols LK, Barone R, Jamar F, Bakker WH, Kwekkeboom DJ, Bouterfa H, Krenning EP (2006). Survival and response after peptide receptor radionuclide therapy with [90Y-DOTA0,Tyr3]octreotide in patients with advanced gastroenteropancreatic neuroendocrine tumors. Semin Nucl Med.

[78] van Essen M, Krenning EP, Bakker WH, de Herder WW, van Aken MO, Kwekkeboom DJ (2007). Peptide receptor radionuclide therapy with 177Lu-octreotate in patients with foregut carcinoid tumours of bronchial, gastric and thymic origin. Eur J Nucl Med Mol Imaging.

[79] Vinjamuri S, Gilbert TM, Banks M, McKane G, Maltby P, Poston G, Weissman H, Palmer DH, Vora J, Pritchard DM, Cuthbertson DJ (2013). Peptide receptor radionuclide therapy with (90)Y-DOTATATE/(90)Y-DOTATOC in patients with progressive metastatic neuroendocrine tumours: assessment of response, survival and toxicity. Br J Cancer.

[80] Werner RA, Lapa C, Ilhan H, Higuchi T, Buck AK, Lehner S, et al. Survival prediction in patients undergoing radionuclide therapy based on intratumoral somatostatin-receptor heterogeneity. Oncotarget. 2017;8(4, 7039):–7049. 10.18632/oncotarget.12402. 10.18632/oncotarget.12402 PMC5351689 27705948

[81] Werner RA, Ilhan H, Lehner S, Papp L, Zsótér N, Schatka I, Muegge DO, Javadi MS, Higuchi T, Buck AK, Bartenstein P, Bengel F, Essler M, Lapa C, Bundschuh RA (2019). Pre-therapy Somatostatin receptor-based heterogeneity predicts overall survival in pancreatic neuroendocrine tumor patients undergoing peptide receptor radionuclide therapy. Mol Imaging Biol.

[82] Yalchin M, Oliveira A, Theocharidou E, Pencharz D, Navalkissoor S, Quigley AM, Walker M, Caplin M, Toumpanakis C (2017). The impact of radiological response to peptide receptor radionuclide therapy on overall survival in patients with metastatic Midgut neuroendocrine tumors. Clin Nucl Med.

[83] Zovato S, Kumanova A, Demattè S, Sansovini M, Bodei L, Di Sarra D, Casagranda E, Severi S, Ambrosetti A, Schiavi F, Opocher G, Paganelli G (2012). Peptide receptor radionuclide therapy (PRRT) with 177Lu-DOTATATE in individuals with neck or mediastinal paraganglioma (PGL). Horm Metab Res.

